# Parameter estimation of a model describing the human fingers

**DOI:** 10.1049/htl2.12070

**Published:** 2023-12-26

**Authors:** Panagiotis Tsakonas, Evans Neil, Joseph Hardwicke, Michael J. Chappell

**Affiliations:** ^1^ School of Engineering University of Warwick Coventry UK; ^2^ Institute of Applied & Translational Technologies in Surgery University Hospitals Coventry and Warwickshire NHS Trust Coventry UK

**Keywords:** biomechanics, Hilbert transforms, image motion analysis, kinematics, motion estimation

## Abstract

The goal of this paper is twofold: firstly, to provide a novel mathematical model that describes the kinematic chain of motion of the human fingers based on Lagrangian mechanics with four degrees of freedom and secondly, to estimate the model parameters using data from able‐bodied individuals. In the literature there are a variety of mathematical models that have been developed to describe the motion of the human finger. These models offer little to no information on the underlying mechanisms or corresponding equations of motion. Furthermore, these models do not provide information as to how they scale with different anthropometries. The data used here is generated using an experimental procedure that considers the free response motion of each finger segment with data captured via a motion capture system. The angular data collected are then filtered and fitted to a linear second‐order differential approximation of the equations of motion. The results of the study show that the free response motion of the segments is underdamped across flexion/extension and ad/abduction.

## Graphical abstract

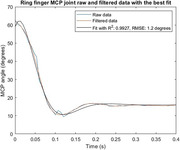

## 1 INTRODUCTION

Mathematical models are crucial in supporting our understanding of physical processes. To be effective, a mathematical model must be able to accurately describe real‐life observations and be able to make robust and reliable predictions of yet unknown events. Furthermore, the assumptions used in the modelling must be clearly indicated and accounted for in the model and analysis. In the literature there are a wide variety of mathematical models that describe the kinematics of human fingers. The most influential models of the biomechanics of the upper extremity are presented in [1, 2, 3, 4, 5, 6]. These models have been extremely helpful in gaining an understanding of the motion of the finger segments and in providing results on the underlying forces of the musculature from experimental procedures. However, in these models, there is no discussion of subject‐specific scaling of the parameters that influence the motion. To allow a mathematical model that describes the motion of the human fingers to scale with different anthropometries, an informed decision on the geometry of the fingers is required, which allows the determination of the mass of each segment. Moreover, the finger segments rotate about their respective joints; hence the determination of the individual masses and subsequent moments of inertia are important factors. In the literature, there are only a few papers that discuss the respective moments of inertia of the finger segments [7, 8]. However, in these papers, the equations used to obtain the moments of inertia are not included. Furthermore, the passive moment generated at each segment due to the passing tendons, synovial fluid, surrounding tissue etc. is assumed to be the same for everyone, and the values assigned to the constants describing this interaction are not referenced [4]. The goal of this paper is therefore to use existing knowledge to further the development of mathematical models that describe the motion of the human finger segments, subject to different anthropometries, and to perform parameter estimation to determine the constants that describe the passive moment generated at each segment. A preliminary version of this work was reported at the BioMedEng 22 conference [9].

## 2 MODELLING

**FIGURE 1:**
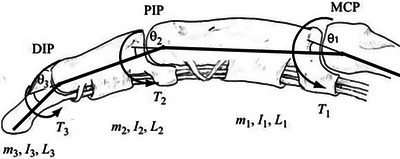
Conceptual image of a finger showing the three degrees of freedom in flexion/extension. $\theta_{1},\;\theta_{2},\;\theta_{3}$ correspond to the flexion/extension angle of the proximal, middle, and distal segments, respectively. $m_{i},I_{i},\;L_{i},\;T_{i}$ corresponds to the mass, the moment of inertia, the length, and the overall torque of segment i. IReproduced with permission.^[^ ^11^ ^]^

The equations of motion for human finger segments are established as a serial linkage of three compound cylindrical rod pendula with a density of $\rho \;=\;1.16\;\mathrm{gr}/{\mathrm{cm}}^{3}$ [10]. Applying Lagrangian mechanics with four degrees of freedom—three in the flexion/extension of the metacarpophalangeal (MCP), proximal interphalangeal (PIP), and distal interphalangeal (DIP) joints, and one in the adduction/abduction of the MCP joint—in a similar manner to the approach adopted in [1]. A spherical coordinate system is used, with its origin attached to the MCP joint. The elevation angle corresponds to the flexion/extension motion of the segments, with its positive direction being perpendicular to the dorsal side of the palm facing away from it and the azimuthal direction to the ad/abduction. Figure 1 shows the flexion/extension angles in their respective frame of reference alongside the mass, moment of inertia, segment length and overall torque at each joint.

**FIGURE 2:**
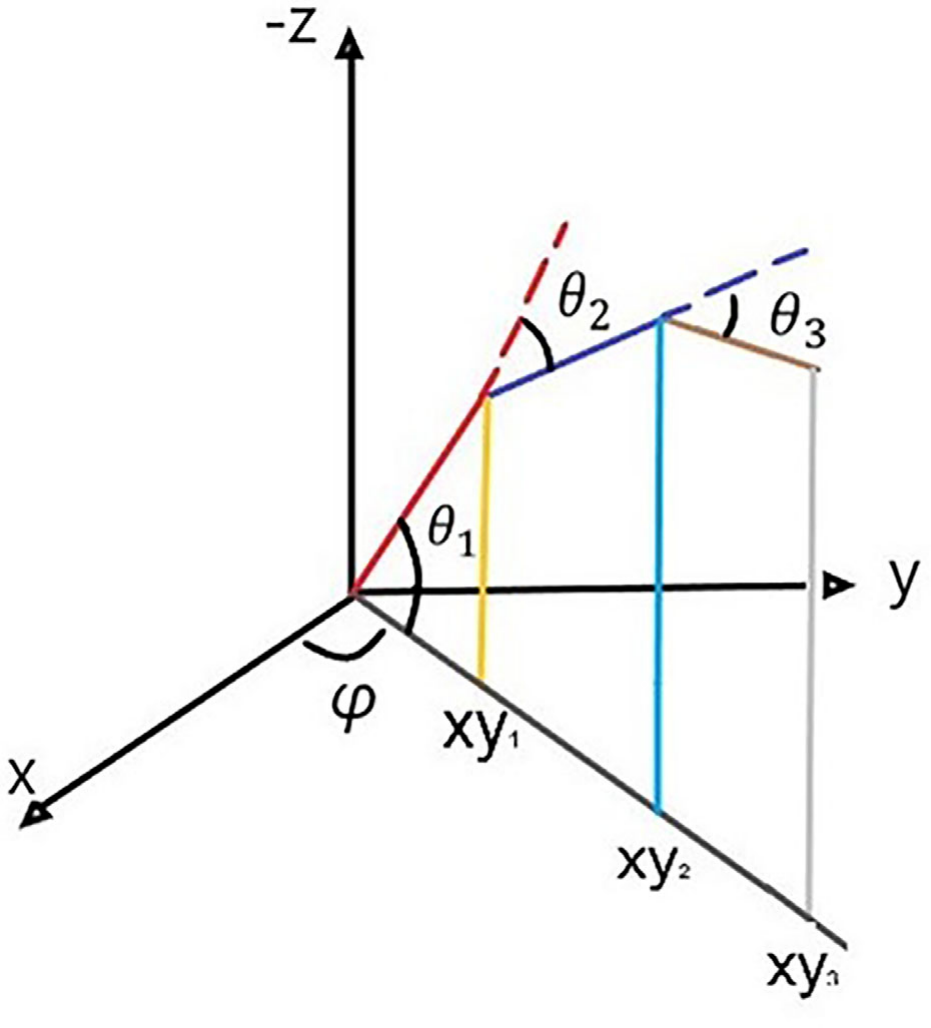
The spherical coordinate system used in the modelling. Angles $\theta_{1},\theta_{2},\theta_{3}$ corresponds to the flexion angles of the MCP, PIP and DIP joints, respectively. $xy_{1},\;xy_{2},\;xy_{3}$ are the azimuthal projections of each end point. Angle ϕ is the abduction angle of each finger.

The convention used in this present model is that when all segments are fully extended, the elevation angle of the respective local reference frame is zero. The set of equations describing the system as a linkage of three rigid cylindrical rod pendula for the midpoint of each cylinder is shown in (1). Figure 2 shows the spherical coordinate system used in our model.

The variables in (1) are functions of time, but the time notation is omitted for convenience. Let $\theta_{i},m_{i},L_{i},\phi$ denote the flexion angle, mass, length of segment *i* (*i* = 1 to 3) and the corresponding abduction angle, respectively. Then the equations describing the position of the finger segments in the given spherical coordinate system are given by:

(1) $$\left\{\begin{array}{c} \;z_{1}=\;-\frac{L_{1}}{2}sin\left(\theta_{1}\right)\\ x\;y_{1}=\;-\frac{L_{1}}{2}cos\left(\theta_{1}\right)\\ \;z_{2}=\;-\left(L_{1}\sin\left(\theta_{1}\right)+\frac{L_{2}}{2}\sin\left(\theta_{2}\right)\right)\\ x\;y_{2}=\;-\left(L_{1}\cos\left(\theta_{1}\right)+\frac{L_{2}}{2}\cos\left(\theta_{2}\right)\right)\\ \;z_{3}=\;-\left(L_{1}\sin\left(\theta_{1}\right)+L_{2}\sin\left(\theta_{2}\right)+\frac{L_{3}}{2}\sin\left(\theta_{3}\right)\right)\\ x\;y_{3}=-\left(L_{1}\;\cos\left(\theta_{1}\right)+L_{2}\cos\left(\theta_{2}\right)+\frac{L_{3}}{2}cos\left(\theta_{3}\right)\right)\\ \;\\ \;x_{1}=\;xy_{1}\cos\left(\phi \right)\\ \;y_{1}=\;xy_{1}\sin\left(\phi \right)\\ \;x_{2}=\;xy_{2}\cos\left(\phi \right)\\ \;y_{2}=\;xy_{2}\sin\left(\phi \right)\\ \;x_{3}=\;xy_{3}\cos\left(\phi \right)\\ \;y_{3}=\;xy_{3}\sin\left(\phi \right) \end{array}\right.$$

In (1) $z_{i}$ is the Cartesian representation of the projection of each point into the elevation plane, $xy_{i}$ is the projection of each point in the azimuthal plane and $x_{i},\;y_{i}$ are the individual Cartesian coordinates of the $xy_{i}$ vector (all for *i* = 1 to 3).

The moment of inertia, $I_{o,i}$, of a uniform cylinder, of mass $m_{i}$, length $L_{i}$ and radius $R_{i}$, about its midpoint and the calculation of the mass of each segment are shown in (2) [12]

(2) $$\left\{\begin{array}{c} \;I_{o,i}=m_{i}\;\left(\frac{R_{i}^{2}}{4}+\frac{L_{i}^{2}}{12}\right)\\ \;m_{i}=\;\pi \rho R_{i}^{2}L_{i} \end{array}\right.$$

The total kinetic energy of the system is given by (3):

(3) $$K_{\mathrm{total}}=\frac{1}{2}\left(\sum_{i=1}^{3}m_{i}\left({\dot{z}}_{i}^{2}+{\dot{x}}_{i}^{2}+{\dot{y}}_{i}^{2}\right)+I_{o,i}\left({\dot{\theta }}_{i}^{2}+{\dot{\phi }}^{2}\cos^{2}\theta_{i}\right)\right)$$

The gravitational potential energy is given by (4):

(4) $$V_{\mathrm{grav}}=\sum_{i=1}^{3}m_{i}gz_{i}.$$

In the literature, there are papers that include a passive moment generated at each joint from the surrounding tissue, tendons and synovial fluid and this has been modelled as a linear torsional spring and damper [4, 8, 13]. The torsional spring effect arises in the potential energy term, as shown in (5):

(5) $$V_{\mathrm{spring}}=\frac{1}{2}\;\left(\sum_{i\;=\;1}^{3}\left[K_{i}\;{\left(\theta_{i}-\theta_{eq,i}\right)}^{2}\right]+K_{a}{\left(\phi -\phi_{eq}\right)}^{2}\right)$$

where $K_{i},K_{a},\theta_{eq,i},\phi_{eq}$ are: the torsional spring constant for the flexion/extension movement of segment *i*, the torsional spring constant for the abduction movement, the equilibrium angles of the flexion/extension of segment *i* and ad/abduction movements that correspond to the functional position of the hand, respectively [14].

The Lagrangian is expressed as the difference between the kinetic and potential energy of the system, that is,

(6) $$L\;=K_{\mathrm{total}}\;-\left(V_{\mathrm{grav}}+V_{\mathrm{spring}}\right).$$

Lastly, the torsional damper effects are introduced into the Lagrangian as a Rayleigh dissipation function [15]:

(7) $$R=\frac{1}{2}\left(\sum_{i=1}^{3}\left[B_{i}{\dot{\theta }}_{i}^{2}\right]+B_{a}{\dot{\phi }}^{2}\right)$$

where *B_i_*, ${\dot{\theta }}_{i}$, *B_a_*, $\dot{\phi }$ are the torsional damper constants of the flexion/extension and the flexion/extension angular velocity of segment *i*, and the torsional damper constant and velocity for the ad/abduction movement respectively of segment *i*.

Let $q\;=\;\{\theta_{1},\theta_{2},\theta_{3},\phi \}$ denote the vector of the generalized coordinates, then the equations of motion for each degree of freedom are obtained via the Euler–Lagrange equations derived using the D'Alembert principle of virtual work [16] and given by:

(8) $$\frac{d}{dt}\left(\frac{\partial L}{\partial {\dot{q}}_{\iota }}\right)-\frac{\partial L}{\partial q_{i}}+\frac{\partial R}{\partial {\dot{q}}_{\iota }}=T_{i}$$

Here $T_{i}$ corresponds to the sum of the torques that is present during motion. During free movement, these are the muscle torques exerted at each degree of freedom and these are of the form:

(9) $$T_{i\;}=\sum_{j}F_{j}\frac{\partial r_{j}}{\partial q_{i}}$$

where $F_{j}$ is the muscle force production which is assumed to be based on a Hill‐muscle model formulation, and the partial derivative corresponds to the moment arm of muscle *j* that spans the joint of segment *i*, with *r* being the musculotendon length as a function of the generalized coordinates [4, 17, 18]. For the ad/abduction movement the muscles that span the MCP joint are considered as actuators.

From (8), the well‐known form of the equations of motion as shown in [4] is obtained. The main difference between the two models is that in the one presented here, an informed approximation of the geometry of the finger segments is used, which allows for a consistent determination of the moment of inertia of each segment based on the anthropometry of the individual. An approximation of the equations of motion for each degree of freedom can be obtained that can be solved analytically. This approximation is called the IBK approximation, where *I* denotes the moment of inertia, *B* the torsional damper and *K* the torsional spring constants respectively. In the literature, this type of model has been utilised to determine the spring and damper constants for different types of movements [19, 20, 21]. The linear approximation of (8) is shown in (10) below:

(10) $$I_{i}{\ddot{q}}_{i}+B_{i}{\dot{q}}_{\iota }+K_{i}\left(q_{i}-q_{eq,i}\right)=T_{i}$$

where $I_{i},B_{i},K_{i}$ are the moment of inertia, the torsional damper and the torsional spring constants for each degree of freedom.

Before performing model parameter estimation, a structural identifiability analysis is required to determine whether all unknown parameters in the model can be identified uniquely from the given model observations/measurements [22]. One important remark to note that will be useful in the identifiability analysis, is that the moment of inertia is uniquely determined from the first term of (8) for each degree of freedom. By measuring the length and radius of each finger segment, one can obtain an analytical expression for estimating its theoretical moment of inertia. The equations that derive the theoretical moment of inertia for the proximal (i=1), middle (i=2), and distal (i=3) segments and for the abduction movement (i=a) are given below, respectively, as follows:

(11) $$I_{1}=m_{1}\;\left(\frac{R_{1}^{2}}{4}+\frac{L_{1}^{2}}{3}\right)+L_{1}^{2}\left(m_{2}+m_{3}\right)$$

(12) $$I_{2}=m_{2}\;\left(\frac{R_{2}^{2}}{4}+\frac{L_{2}^{2}}{3}\right)+L_{2}^{2}m_{3}$$

(13) $$I_{3}=m_{3}\;\left(\frac{R_{3}^{2}}{4}+\frac{L_{3}^{2}}{3}\right)$$

(14) $$\begin{array}{ccc} I_{a} & = & I_{1}\;\cos^{2}\left(\theta_{1}\right)+I_{2}\cos^{2}\left(\theta_{2}\right)+I_{3}\cos^{2}\left(\theta_{3}\right)\\ & & +L_{1}L_{2}\left(m_{2}+2m_{3}\right)\cos\left(\theta_{1}\right)\cos\left(\theta_{2}\right)\\ & & +L_{3}m_{3}\left(L_{1}\cos\left(\theta_{1}\right)\cos\left(\theta_{3}\right)+L_{2}\cos\left(\theta_{2}\right)\cos\left(\theta_{3}\right)\right) \end{array}$$

## 3 STRUCTURAL IDENTIFIABILITY

Structural identifiability analysis is concerned with the question of whether the parameters of a model can be uniquely determined in the theoretical situation of noise‐free, continuous measurement/observation data [22]. Regardless of the complexity of a model, if it is shown that the model is unidentifiable then it is not possible to determine the model parameters uniquely or locally, that is, there is an (uncountably) infinite set of model parameter values that can give rise to the same input‐output problem. It is therefore important to undertake an identifiability analysis on the model of (10) as a prerequisite to estimating the parameters of the model and to support the design of an appropriate experimental procedure to ensure parameter identifiability for the given model outputs.

The free response of (10) is chosen as the model for our parameter estimation that corresponds to the loading/unloading of the torsional spring component. In real‐life this is equivalent to the following type of motion:

1. Participants keep their hands in a relaxed position with no active movement or external force applied.
2. The researcher moves each segment to an arbitrary position within its range of motion.
3. The segment is then released.
4. The response of the segment to its return to its natural steady state is recorded.

The structural identifiability analysis of the free response of (10) is determined using the Laplace transform approach [23]. Dividing by the moment of inertia in (10), its free response is then given by:

(15) $${\ddot{q}}_{i}+\frac{B_{i}}{I_{i}}{\dot{q}}_{\iota }+\frac{K_{i}}{I_{i}}\left(q_{i}-q_{eq,i}\right)=0$$

The initial conditions for each generalised coordinate will be the random initial angle within the range of motion of each segment $q_{0,i}$ and a zero initial angular velocity. Taking the Laplace transform of (14) with these initial conditions yields the following Laplace transform of the response:

(16) $$Q_{i}\;\left(s\right)=\frac{q_{0,i}\left(s^{2}+\frac{B_{i}}{I_{i}}\;s\right)+\frac{q_{eq,i\;}K_{i}}{I_{i}}}{s\left(s^{2}+\frac{B_{i}}{I_{i}}s+\frac{K_{i}}{I_{i}}\right)}\;$$

From [23], each coefficient of the individual powers of 𝑠 in the numerator and denominator of (16) is uniquely determined if and only if, the coefficient of the highest power of 𝑠 in the denominator is one. From (16), the given response function coefficients (moment invariants) that can be uniquely determined are as follows:

(17) $$q_{0,i},\;q_{eq,i},\frac{B_{i}}{I_{i}},\frac{K_{i}}{I_{i}}$$

The model is therefore structurally unidentifiable since only the parameter combinations $K_{i}/I_{i}$ and $B_{i}/I_{i}$ are identifiable (can be uniquely determined) and not the individual parameters within these combinations. *A‐priori* knowledge of one parameter is therefore required for all of the remaining parameters to be uniquely determined. Recall that the parameter $I_{i}$ for the moment of inertia can be estimated from (11–14) for each segment. Thus, if the moment of inertia $I_{i}$ is known, then the remaining two parameters $K_{i}$ and $B_{i}$ can be determined uniquely for each segment from the given input‐output relationship.

## 4 METHODS

The free response of each finger segment (index through little fingers) in the four degrees of freedom model was studied. As mentioned previously, the free response movement corresponds to the involuntary motion of each segment, which is attributed to the loading/unloading of the torsional spring component of the passive moment. The free response movements of the thumb are not discussed in this paper. Participants were instructed to hold their forearm at a 90° angle with respect to their arm. For the flexion movement, the participant's wrist was in its neutral position. For the abduction movement, their wrist was rotated to 90° of pronation. For that wrist orientation, gravitational contributions are neglected since gravity is assumed to be perpendicular to the plane of motion. For the abduction movement participants were asked to keep their individual segments at full extension (all flexion angles set to zero), because the moment of inertia of the abduction movement is dependent on the individual segment flexion angles, as shown in (14). Throughout the trials, participants were instructed to have their fingers relaxed and not to apply any voluntary resistance. Great care was taken to reduce the movement of the proximal segments, minimizing the contributions of the Coriolis forces during the free response movement.

**FIGURE 3:**
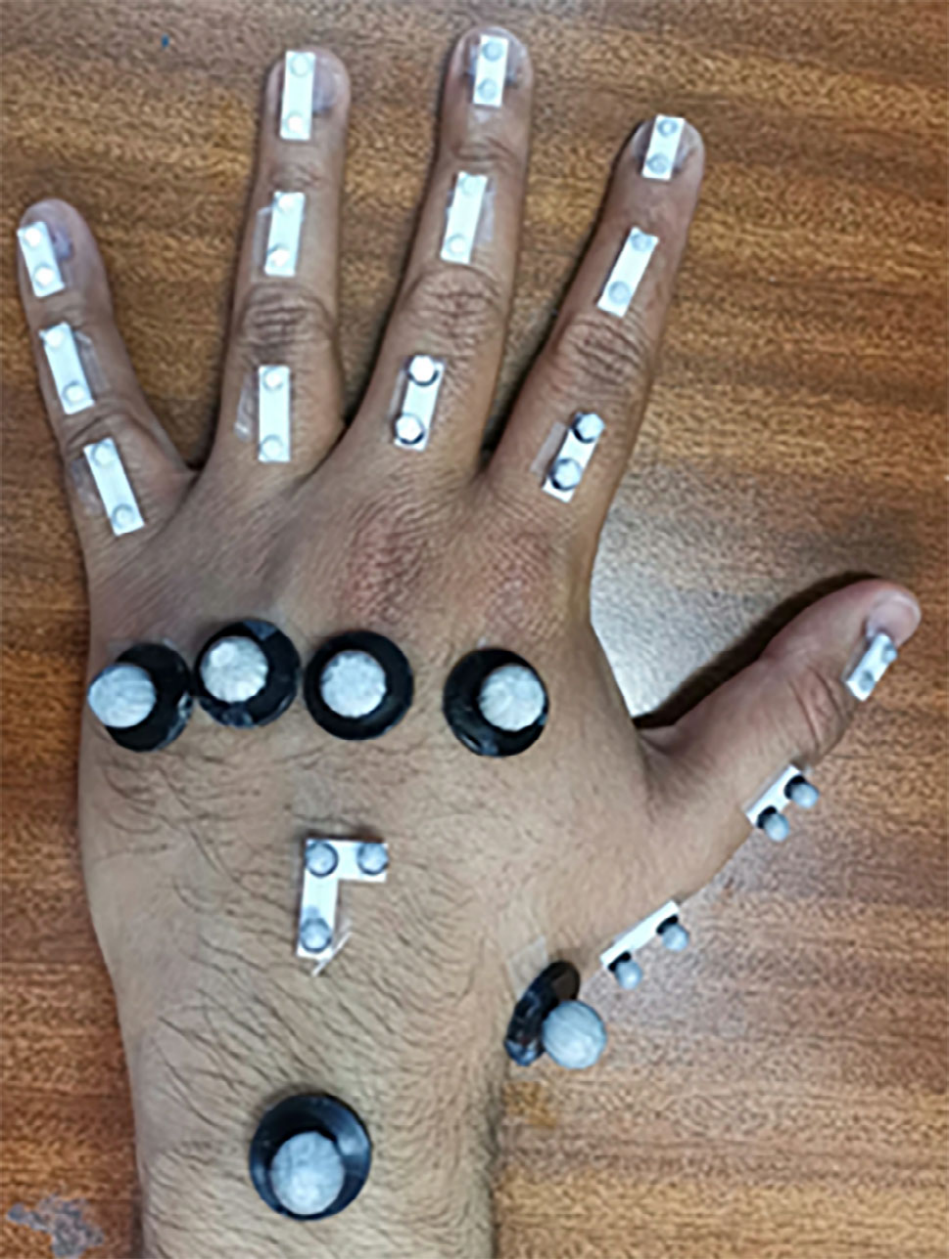
Marker set developed for determining flexion/extension and ad/abduction angles from able‐bodied participants.

A novel marker set was developed, as shown in Figure 3 that allows for the determination of the segment angles from motion capture data. Segment angles were determined using vector calculus. Each segment is represented by a vector between the proximal and distal markers for the joint. A vector between the mid‐ distance of the radial and ulnar styloid processes (the marker shown with a red arrow in Figure 3) and the distal marker to the MCP joint of each finger (shown with yellow and white arrows in Figure 3 is also used to determine the flexion/extension angle of each proximal segment.) Ad/abduction angles are determined between the vector defining the middle of the palm (mid‐distance between ulnar and radial styloid marker (red arrow) to the distal marker of the middle segment's MCP joint (white arrow)) and the vector defined by the mid distance between the ulnar and the radial styloid marker (red arrow) and the proximal marker on the proximal segment (green arrows) of each finger. The L‐shaped marker is used to define the anatomical planes of the dorsal side of the palm. Each vector is projected onto the sagittal (flexion/extension) or radioulnar (ad/abduction) planes and the dot product between the tail of the distal vector translated to the tail of the proximal vector, with respect to the joint of interest, is used to determine angle [24]. Angle extraction from the motion capture data was performed in Vicon Nexus Procalc [24].

At least 15 trials were recorded per degree of freedom per finger. In total, each participant had at least 240 trials for the index to the little finger segments. Before marking the participant, the length and diameter of all their segments were measured and these values were used to determine the mass and moments of inertia of each segment. Motion capture data were recorded at 150 Hz using 12 infrared cameras in the Motion Capture Laboratory within the School of Engineering at the University of Warwick. A total of 23 able‐bodied participants were recruited for the study. The exclusion criteria for the study were participants that had been diagnosed with arthritis, or had a digit amputation, or who were less than 18 years old. Written informed consent was taken before the experimental procedure. This study was granted full approval from the Biomedical and Scientific Research Ethics Committee (BSREC) at the University of Warwick (ref. Number: BSREC 55/21‐22), on 15/03/2022.

**FIGURE 4:**
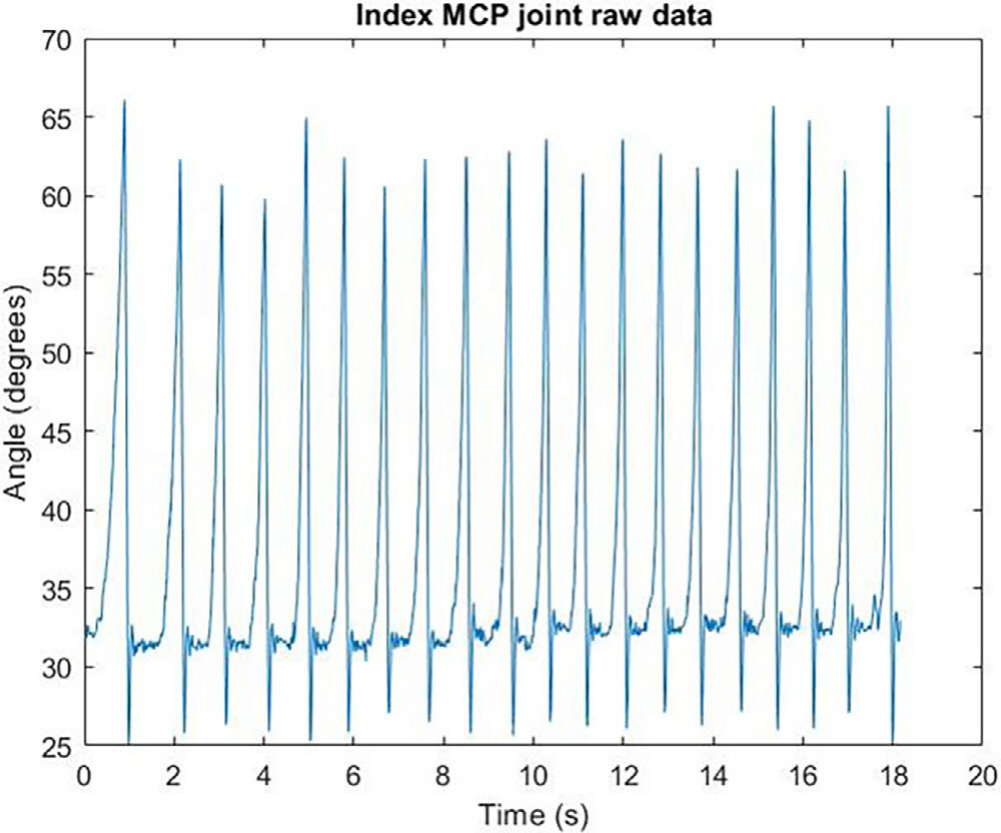
Index finger MCP joint movement raw data. The characteristic response of the free response movement where the finger segment returns to its steady state can be seen.

**FIGURE 5:**
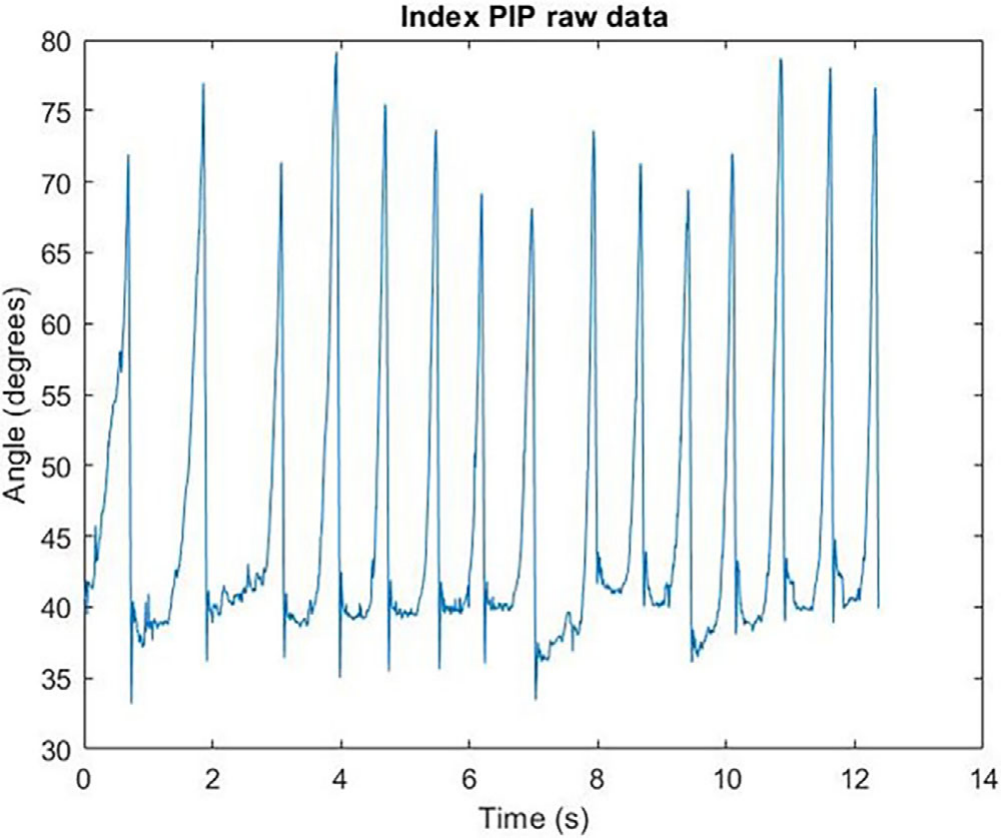
Index finger PIP joint movement raw data. The characteristic response of the free response movement where the finger segment returns to its steady state can be seen.

**FIGURE 6:**
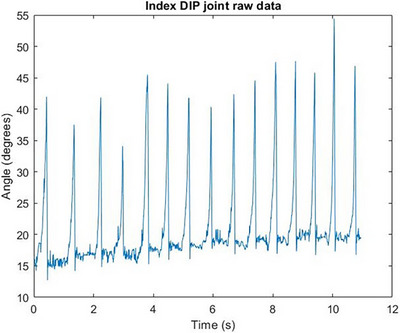
Index finger DIP joint raw data. The characteristic response of the free response movement where the finger segment returns to its steady state can be seen.

**FIGURE 7:**
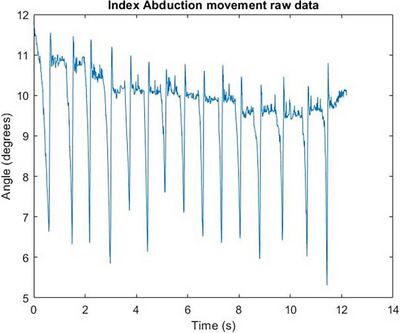
Index finger abduction movement raw data. The characteristic response of the free response movement where the finger segment returns to its steady state can be seen.

**FIGURE 8:**
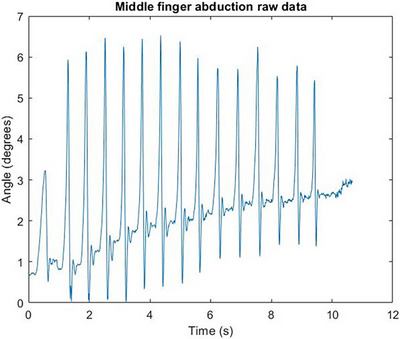
Middle finger abduction movement raw data. The characteristic response of the free response movement where the finger segment returns to its steady state can be seen.

In Figure 4, the characteristic response of the free response movement where the finger segment returns to its steady state can be seen. In Figure 5, the characteristic response of the free response movement where the finger segment returns to its steady state can be seen. In Figure 6, the characteristic response of the free response movement where the finger segment returns to its steady state can be seen. In Figure 7, the characteristic response of the free response movement where the finger segment returns to its steady state can be seen. In Figure 8, the characteristic response of the free response movement where the finger segment returns to its steady state can be seen. The raw data of the free response of each degree of freedom can be seen.

For all fingers, except the middle finger, the abduction‐free response movement of the whole digit was performed by moving each finger towards the middle one. This is evident from Figure 7, where the free response movement data are inverted compared to the data shown in Figure 8, where the middle finger was moved away from the midline of the palm.

**FIGURE 9:**
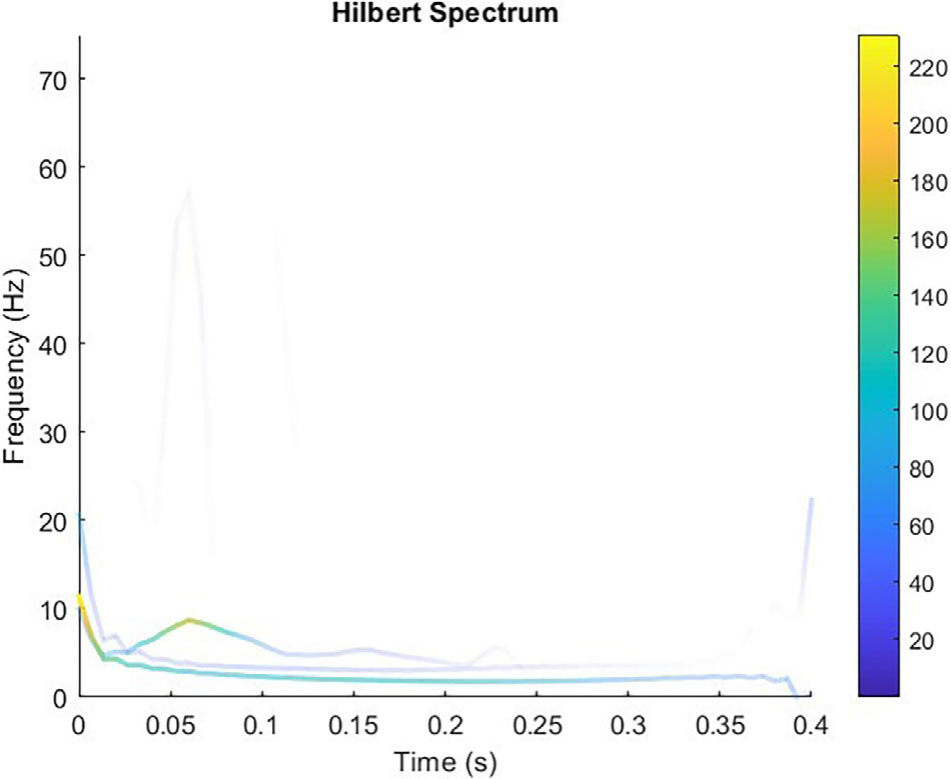
Hilbert–Huang spectrum of a single trial of the proximal segment of a ring finger from one of the participants. The colour coded lines indicate the instantaneous energy at each point of the intrinsic mode function. The highest frequency that also has the highest instantaneous energy is 11.35 Hz.

The angular data extracted from Procalc, were low‐pass filtered using a fourth order Butterworth filter with a cut‐off frequency of 15 Hz for the MCP and PIP joint movements and 12 Hz for the DIP joint and abduction movements. The cut‐off frequencies were determined by plotting the Hilbert–Huang spectrum of randomly selected trials of the signal and averaging the highest frequencies that had the highest instantaneous energies, as shown in Figure 9. Instantaneous energy is proportional to the amplitude of the transformed signal and has units of (rad^2^). Consecutively, the peaks of the data were identified, and the model was fitted from the onset of the peak up to its steady state, where the natural response motion of the finger segments occurs.

**FIGURE 10:**
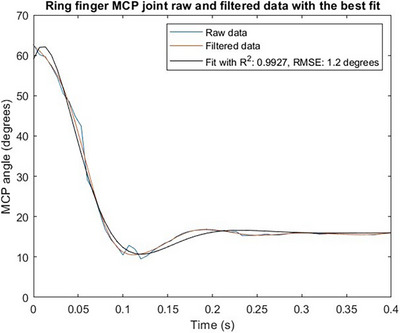
Raw and filtered data of the MCP joint movement of the ring finger of participant, alongside the best fit to the filtered data. The fit to the filtered data has a RMSE of 1.2° and a ***R*** ^2^ value of 0.9927 suggesting an excellent fit.

Figure 10 shows the raw and filtered data alongside the fit with the lowest root mean square error for the same trial.

The filtered data were then fitted to the analytical expressions for the three types of motion of the free response underdamped (18–19), critically damped (20–21), and overdamped (22–23).

## 5 UNDERDAMPED MOTION

The underdamped motion of the free response is achieved when the discriminant of the characteristic polynomial of the denominator of (16) is negative. The resulting analytic expression is shown below:

(18) $$\left\{\begin{array}{c} \begin{array}{c} q_{i}\;\left(t\right)=q_{eq,i}\;+Ae^{-at}\left(C\sin\left(\omega t\right)+\cos\left(\omega t\right)\right)\\ \omega \;=\frac{\sqrt{4K_{i}I_{i}-B_{i}^{2}}}{2I_{i}}\; \end{array}\\ a\;=\frac{B_{i}}{2I_{i}}\; \end{array}\right.$$

By fitting the analytic expression in (18) to the raw data, the unknown model parameters can then be determined from the following expressions:

(19) $$\left\{\begin{array}{c} \;B_{i}=\;2aI_{i}\\ \;K_{i}=I_{i}\;\omega^{2}+\frac{B_{i}^{2}}{4I_{i}}\; \end{array}\right.$$

## 6 CRITICALLY DAMPED MOTION

The critically damped motion of the free response is achieved when the discriminant of the characteristic polynomial of the denominator of (16) is zero. The resulting analytic expression is shown below.

(20) $$\left\{\begin{array}{c} q_{i}\;\left(t\right)=q_{eq,i\;}\;+Ae^{-at}\left(at+1\right)\\ a\;=\frac{B_{i}}{2I_{i}}\;\\ B_{i}^{2}-4K_{i}\;I_{i}=\;0 \end{array}\right.$$

By fitting the analytic expression of (20) to the raw data the unknown model parameters can then be determined from the following expressions:

(21) $$\left\{\begin{array}{c} \;B_{i}=\;2aI_{i}\\ \;K_{i}=\frac{B_{i}^{2}}{4I_{i}}\; \end{array}\right.$$

## 7 OVERDAMPED MOTION

The overdamped motion of the free response is achieved when the discriminant of the characteristic polynomial of the denominator of (16) is positive. Let a,b be the roots of the corresponding characteristic polynomial with a>b. The resulting analytic expression is shown below:

(22) $$\left\{\begin{array}{c} q_{i}\;\left(t\right)=q_{eq,i\;}\;+Ae^{at}+Ce^{bt}\\ b+a\;=\;-\frac{B_{i}}{I_{i}}\\ b*a\;=\frac{K_{i}}{I_{i}}\;\\ a,b<0 \end{array}\right.$$

Since a,b are the roots of a second order polynomial then the parameters can be estimated from (22) using:

(23) $$\left\{\begin{array}{c} \;B_{i}=\;-\left(b+a\right)I_{i}\\ \;K_{i}=\;b*a*I_{i} \end{array}\right.$$

A custom MATLAB code was written that performed the above‐mentioned steps. The parameters were extracted for the fit that had the lowest root mean square error. For each segment, a mean and standard deviation for each parameter were obtained. Any missing parameters from the tables below were due to participants having a non‐disqualifying injury at the specific segment or the number of available trials where the markers are shown being fewer than five.

## 8 RESULTS

Tables 1, 2, 3, 4 show the mean values of the torsional spring and damper parameter estimates as determined from the analysis above for each segment for all participants, alongside the respective moments of inertia and damping ratios.

**TABLE 1:** Index finger mean parameter estimate values for all degrees of freedom.

Index															
MCP flexion		PIP flexion		DIP flexion		Abduction		Moment of inertia (Kg m^2^/rad)				Damping ratio			
K (Nm/rad)	B (Nms/rad)	K (Nm/rad)	B (Nms/rad)	K (Nm/rad)	B (Nms/rad)	K (Nm/rad)	B (Nms/rad)	Proximal	Middle	Distal	Abduction	Proximal	Middle	Distal	Abduction
0.0482	0.0013	0.0169	5.26 × 10^‐4^	0.002814	8.23 × 10^‐5^	0.098	3.70 × 10^‐3^	3.45 × 10^‐5^	6.33 × 10^‐6^	1.37 × 10^‐6^	7.52038 × 10^‐5^	0.503967	8.03E‐01	6.63E‐01	6.81E‐01
0.0204	7.00 × 10‐4	0.0132	3.65 × 10^‐4^	0.0023	8.42 × 10^‐5^	0.0515	1.70 × 10^‐3^	1.44 × 10^‐5^	5.7 × 10^‐6^	1.28E‐06	4.39698E‐05	0.645271	0.665955	0.776966	5.65E‐01
0.0317	0.0012	0.0149	5.07 × 10^‐4^	0.0013	5.62 × 10^‐5^	0.1259	4.30 × 10^‐3^	2.83 × 10^‐5^	7.47 × 10^‐6^	1.17 × 10^‐6^	6.94145 × 10^‐5^	6.34E‐01	7.60E‐01	0.721578	0.727278061
0.0299	7.02 × 10^‐4^	0.0062	0.00018701	0.0012502	4.16 × 10^‐5^	0.0712	1.30 × 10^‐3^	1.32 × 10^‐5^	3.19 × 10^‐6^	7.3 × 10^‐7^	3.1884 × 10^‐5^	0.558089	6.65E‐01	0.687718	4.31E‐01
0.049	1.10 × 10^‐3^	0.0096	2.88 × 10^‐4^	0.002	4.82 × 10^‐5^	0.0802	2.50 × 10^‐3^	2.32 × 10^‐5^	4.78 × 10^‐6^	9.68 × 10^‐7^	5.32165 × 10^‐5^	5.15E‐01	6.72E‐01	5.48E‐01	6.05E‐01
0.028683	9.61 × 10^‐4^	0.0053766	0.00015258	0.0011042	3.05 × 10^‐5^	0.0502	1.60 × 10^‐3^	1.4 × 10^‐5^	2.62 × 10^‐6^	3.81 × 10^‐7^	2.90447 × 10^‐5^	0.75833	0.643175	0.743493	0.662528279
0.032036	0.00078545	0.011965	0.00026433	0.0021662	5.56 × 10^‐5^	0.0924	0.0022	1.86 × 10^‐5^	3.7 × 10^‐6^	6.83 × 10^‐7^	4.1098 × 10^‐5^	5.08E‐01	6.28E‐01	7.23E‐01	5.64E‐01
0.027898	0.00068866	0.008704	0.00025064	0.0019662	4.88 × 10^‐5^	0.0787	1.80 × 10^‐3^	1.52 × 10^‐5^	3.6 × 10^‐6^	6.79 × 10^‐7^	3.55902 × 10^‐5^	0.528168	0.707492	0.66783	0.537761618
0.0241	0.00063765	0.0102	2.49 × 10^‐4^	0.0035392	7.94 × 10^‐5^	0.1091	3.00 × 10^‐3^	1.71 × 10^‐5^	4.2 × 10^‐6^	1.05 × 10^‐6^	4.27133 × 10^‐5^	4.96E‐01	6.01E‐01	0.649928	0.694859876
0.039058	0.0013767	0.016351	0.00046946	0.0046	1.07 × 10^‐4^	0.1709	3.40 × 10^‐3^	2.8 × 10^‐5^	6.06 × 10^‐6^	1.43 × 10^‐6^	6.58132 × 10^‐5^	0.658034	0.745797	0.657193	0.506899021
0.02784	0.00075546	0.0101	1.98 × 10^‐4^	0.0019	4.65 × 10^‐5^	0.1208	2.90 × 10^‐3^	1.71 × 10^‐5^	5.82 × 10^‐6^	1.02 × 10^‐6^	4.70887 × 10^‐5^	0.547745	0.407724	0.528263	0.607961688
0.0527	0.0015	0.0125	3.01 × 10^‐4^	0.0024	5.16 × 10^‐5^	0.1463	3.40 × 10^‐3^	2.9 × 10^‐5^	6.49 × 10^‐6^	1.28 × 10^‐6^	6.68595 × 10^‐5^	0.607145	0.527752	0.466363	0.543557243
0.050911	0.0013044	0.018981	4.64 × 10^‐4^	0.0048153	0.00013878	0.0995	4.50 × 10^‐3^	3.17 × 10^‐5^	7.29 × 10^‐6^	2.12 × 10^‐6^	7.97117 × 10^‐5^	0.513391	0.62442	0.686762	0.798932128
0.045774	0.0012152	0.014418	0.00040187	0.003	7.98 × 10^‐5^	0.1823	4.60 × 10^‐3^	2.71 × 10^‐5^	5.84 × 10^‐6^	1.02 × 10^‐6^	6.08529 × 10^‐5^	0.545099	0.692298	0.720179	0.690548194
0.037514	0.0012874	0.014673	0.00043554	0.004	9.47 × 10^‐5^	0.1382	4.10 × 10^‐3^	2.6 × 10^‐5^	7.31 × 10^‐6^	1.49 × 10^‐6^	6.67427 × 10^‐5^	0.652066	0.664874	0.612276	0.674991083
0.0188	4.26 × 10^‐4^	0.0081263	2.11 × 10^‐4^	0.0018	5.03 × 10^‐5^	0.0789	1.60 × 10^‐3^	1.2 × 10^‐5^	3.74 × 10^‐6^	7.64 × 10^‐7^	3.23656 × 10^‐5^	0.448844	0.604663	0.678694	0.500621599
0.040299	0.0011178	0.0142	3.76 × 10^‐4^	0.0032	8.71 × 10^‐5^	0.1437	0.0037	2.66 × 10^‐5^	5.93E‐06	1.17 × 10^‐6^	6.18267 × 10^‐5^	0.540097	0.646793	0.711441	0.620662206
0.069522	0.0018435	0.03453	0.00092837	0.0059797	0.00016245	0.223	0.0046	4.66 × 10^‐5^	1.15 × 10^‐5^	2.31 × 10^‐6^	0.000112923	0.512241	7.35E‐01	0.691754	4.58E‐01
0.053867	0.0012284	0.019551	5.68 × 10^‐4^	0.0037502	9.94 × 10^‐5^	0.1673	3.90 × 10^‐3^	3.23 × 10^‐5^	7.65E‐06	1.38 × 10^‐6^	7.61191 × 10^‐5^	4.66E‐01	7.35E‐01	0.691965	5.46E‐01
0.028036	0.00076367	0.013415	0.00036348	0.0020245	7.29 × 10^‐5^	0.0831	1.40 × 10^‐3^	1.73 × 10^‐5^	5.15E‐06	7.04 × 10^‐7^	4.35033 × 10^‐5^	0.548614	6.91E‐01	0.965682	3.68E‐01
0.026818	0.00069555	0.014359	0.00035485	0.0025338	6.58 × 10^‐5^	0.0971	2.00 × 10^‐3^	1.65 × 10^‐5^	4.69E‐06	1.04 × 10^‐6^	4.34315 × 10^‐5^	5.22E‐01	6.83E‐01	6.40E‐01	4.87E‐01
0.031978	0.0011844	0.0258	7.05 × 10^‐4^	0.0053	1.61 × 10^‐4^	0.1465	0.0037	2.54 × 10^‐5^	1.01 × 10^‐5^	2.16 × 10^‐6^	7.7599 × 10^‐5^	0.657728	6.89E‐01	0.752844	5.49E‐01
0.027251	0.00086769	0.016639	0.00049613	0.0030466	9.49 × 10^‐5^	0.0999	0.0019	2.25 × 10^‐5^	7.42E‐06	1.09 × 10^‐6^	5.95085 × 10^‐5^	0.553856	7.06E‐01	8.22E‐01	3.90E‐01

												Mean Damping ratio			
												Proximal	Middle	Distal	Abduction
												0.561734	6.65E‐01	6.87E‐01	5.74E‐01
											Standard deviation	0.074728	8.18E‐02	0.099985	1.10E‐01

**TABLE 2:** Middle finger mean parameter estimate values for all degrees of freedom.

Middle															
MCP flexion		PIP flexion		DIP flexion		Abduction		Moment of inertia (Kg m^2^/rad)				Damping ratio			
K (Nm/rad)	B (Nms/rad)	K (Nm/rad)	B (Nms/rad)	K (Nm/rad)	B (Nms/rad)	K (Nm/rad)	B (Nms/rad)	Proximal	Middle	Distal	Abduction	Proximal	Middle	Distal	Abduction
0.054261	0.0017286	0.0231	6.36 × 10^−4^	0.0037206	1.06 × 10^−4^	0.1779	5.00 × 10^−3^	4.12 × 10^−5^	8.46 × 10^−6^	1.6 × 10^−6^	9.28844 × 10^−5^	0.578285	7.19E‐01	6.90E‐01	6.15E‐01
0.0403	1.10 × 10^−3^	0.0262	6.59 × 10^−4^	0.0036	1.38 × 10^−4^	#N/A	#N/A	2.7 × 10^−5^	9.47 × 10^−6^	2.04 × 10^−6^	7.83925 × 10^−5^	0.527271	0.66153	0.805794	#N/A
0.0394	0.0012	0.0177	6.65 × 10^−4^	0.0029307	8.66 × 10^−5^	0.1262	3.40 × 10^−3^	2.4 × 10^−5^	9.58 × 10^−6^	1.17 × 10^−6^	6.88835 × 10^−5^	0.616844	0.80677	0.739868	0.576582803
0.035275	9.70 × 10^−4^	0.012952	0.00044088	0.0024	5.49 × 10^−5^	#N/A	#N/A	1.67 × 10^−5^	6.67 × 10^−6^	9.28E‐07	4.82756 × 10^−5^	0.632409	0.75012	0.581232	#N/A
#N/A	#N/A	0.012869	0.00039532	0.0020794	6.55 × 10^−5^	0.0884	2.80 × 10^−3^	1.78 × 10^−5^	6.83 × 10^−6^	1.16 × 10^−6^	5.20228 × 10^−5^	#N/A	0.666707	0.667524	0.652837138
0.026336	0.00088358	0.0084	2.74 × 10^−4^	1.00E‐03	4.28 × 10^−5^	0.03282	9.89E‐04	1.44 × 10^−5^	5.17 × 10^−6^	7.82 × 10^−7^	3.98445 × 10^−5^	0.718392	0.656546	0.764664	0.432492639
0.0289	9.67 × 10^−4^	0.010465	0.00025567	0.0024	6.14 × 10^−5^	0.0566	2.00 × 10^−3^	1.75 × 10^−5^	4.34 × 10^−6^	8.09 × 10^−7^	4.21894 × 10^−5^	0.679632	0.599663	0.696577	0.647128069
0.0331	8.51 × 10^−4^	0.012782	0.00027937	0.0015	3.55 × 10^−5^	0.0972	3.40 × 10^−3^	1.78 × 10^−5^	5.73 × 10^−6^	6.69 × 10^−7^	4.5773 × 10^−5^	0.554315	5.16E‐01	0.559944	0.805955797
0.049695	0.0013778	0.0151	4.20 × 10^−4^	0.0027284	6.02 × 10^−5^	0.1748	3.50 × 10^−3^	2.28 × 10^−5^	6.79 × 10^−6^	9.07 × 10^−7^	5.71845 × 10^−5^	0.647627	0.655736	0.604985	0.553513656
0.035762	0.0015003	0.020961	0.00065422	0.0038183	9.61 × 10^−5^	0.0505	2.90 × 10^−3^	2.92 × 10^−5^	8.04 × 10^−6^	1.22 × 10^−6^	7.23688 × 10^−5^	7.34E‐01	0.796706	0.70466	7.58E‐01
0.037176	0.0011111	0.020019	0.00048074	0.0035001	8.42 × 10^−5^	0.1741	3.20 × 10^−3^	2.49 × 10^−5^	7.34 × 10^−6^	1.19 × 10^−6^	6.44689 × 10^−5^	0.577893	0.626933	0.653286	4.78E‐01
0.0579	0.0018	0.02289	0.00066829	0.0022	7.76 × 10^−5^	0.131	4.80 × 10^−3^	3.96 × 10^−5^	1.05 × 10^−5^	1.45 × 10^−6^	9.5226 × 10^−5^	5.94E‐01	6.81E‐01	6.87E‐01	6.80E‐01
0.039906	0.0011759	0.01863	0.00067521	0.002	7.68 × 10^−5^	0.1167	4.00 × 10^−3^	2.5 × 10^−5^	8.46 × 10^−6^	1.31 × 10^−6^	6.86647 × 10^−5^	0.58915	0.85014	0.748593	0.706525635
0.0454	0.0012	0.0223	6.41 × 10^−4^	0.0052	1.18 × 10^−4^	0.1408	3.40 × 10^−3^	2.13 × 10^−5^	8.1 × 10^−6^	1.65 × 10^−6^	6.31921 × 10^−5^	6.10E‐01	7.54E‐01	6.37E‐01	5.70E‐01
0.045799	0.0016998	0.012197	0.00034959	#N/A	#N/A	0.1634	4.50 × 10^−3^	2.67 × 10^−5^	7.82 × 10^−6^	1.29 × 10^−6^	6.80463 × 10^−5^	0.769074	0.565866	#N/A	0.674767602
0.018271	5.11 × 10^−4^	0.0083	2.38 × 10^−4^	0.0021	5.92 × 10^−5^	0.0459	2.70 × 10^−3^	1.15 × 10^−5^	4.41 × 10^−6^	7.59 × 10^−7^	3.37789 × 10^−5^	0.55801	0.621129	0.741148	1.084188531
0.039335	0.0014526	0.017641	0.00049468	0.0028	8.06 × 10^−5^	0.1407	5.08 × 10^−3^	3.29 × 10^−5^	8.77 × 10^−6^	1.48 × 10^−6^	8.1915 × 10^−5^	0.638592	0.628697	0.625537	0.747486006
0.077381	0.0020949	0.043785	0.0011271	0.0060851	0.00017864	0.29773	0.0078124	5.07 × 10^−5^	1.56 × 10^−5^	2.53 × 10^−6^	0.000134008	0.528665	0.682582	0.720365	0.618410944
0.035136	0.0010277	0.019036	0.00058546	0.0033	9.23 × 10^−5^	0.1734	5.60 × 10^−3^	3.39 × 10^−5^	1.05 × 10^−5^	1.48 × 10^−6^	8.77073 × 10^−5^	0.470573	0.654494	0.660238	0.717985301
0.023862	7.97 × 10^−4^	0.020036	0.00050123	0.0028133	7.67 × 10^−5^	0.10958	1.93 × 10^−3^	2.04 × 10^−5^	7.96 × 10^−6^	1.13 × 10^−6^	5.86082 × 10^−5^	0.571254	0.627565	0.681453	0.380984691
0.0159	6.46 × 10^−4^	0.0070375	0.00036493	0.0014	5.13 × 10^−5^	#N/A	#N/A	1.65 × 10^−5^	6.46 × 10^−6^	1.1 × 10^−6^	4.87607 × 10^−5^	0.631195	0.855462	0.654277	#N/A
0.036289	0.0015204	0.021189	0.00071486	0.0056915	0.00014606	0.106	0.003	2.62 × 10^−5^	9.76 × 10^−6^	1.94 × 10^−6^	7.64282 × 10^−5^	0.779715	0.785818	0.694156	0.527001237
0.026933	0.00074778	0.016159	5.27 × 10^−4^	0.0047639	1.10 × 10^−4^	0.0399	0.0012	2.05 × 10^−5^	9.61 × 10^−6^	1.42 × 10^−6^	6.45195 × 10^−5^	0.502618	0.669156	0.670062	0.373954894

												Mean Damping ratio			
												Proximal	Middle	Distal	Abduction
												0.614089	0.68837	0.681262	0.630016105
											Standard deviation	0.151367	0.088259	0.153513	0.264141901

**TABLE 3:** Ring finger mean parameter estimate values for all degrees of freedom.

Ring															
MCP flexion		PIP flexion		DIP flexion		Abduction		Moment of inertia (Kg m^2^/rad)				Damping ratio			
K (Nm/rad)	B (Nms/rad)	K (Nm/rad)	B (Nms/rad)	K (Nm/rad)	B (Nms/rad)	K (Nm/rad)	B (Nms/rad)	Proximal	Middle	Distal	Abduction	Proximal	Middle	Distal	Abduction
0.0519	0.0014	0.0183	5.90 × 10^−4^	0.0037733	1.07 × 10^−4^	0.1638	4.70 × 10^−3^	3.07 × 10^−5^	7.11 × 10^−6^	1.59 × 10^−6^	7.44057 × 10^−5^	0.554637	8.18E‐01	6.94E‐01	6.73E‐01
#N/A	#N/A	0.0125	3.12 × 10^−4^	0.0037565	9.46 × 10^−5^	0.0407	9.42 × 10^−4^	1.23 × 10^−5^	6.28 × 10^−6^	1.25 × 10^−6^	4.22174 × 10^−5^	#N/A	0.556718	0.690842	0.359446809
0.04	0.0013	0.0103	3.98 × 10^−4^	0.0025	5.93 × 10^−5^	0.0868	3.00 × 10^−3^	1.95 × 10^−5^	6.36 × 10^−6^	9.91 × 10^−7^	5.30026 × 10^−5^	0.735511	0.777771	0.595374	0.699331354
0.0184	2.67 × 10^−4^	0.0082	1.70 × 10^−4^	0.0022426	5.89 × 10^−5^	0.0318	9.08 × 10^−4^	8.64 × 10^−6^	4.77 × 10^−6^	8.36 × 10^−7^	2.98034 × 10^−5^	0.334586	0.430967	0.679883	0.46631607
0.0375	1.10 × 10^−3^	0.0177	4.80 × 10^−4^	0.0024565	6.86 × 10^−5^	0.1418	3.10 × 10^−3^	2.02E‐05	7.19 × 10^−6^	1.14 × 10^−6^	5.64576 × 10^−5^	0.632677	0.673253	0.646956	0.547812825
0.021249	0.00059712	0.0075	2.51 × 10^−4^	8.97 × 10^−4^	2.77 × 10^−5^	0.0289	1.20 × 10^−3^	8.95× 10^−6^	3.7 × 10^−6^	5.87 × 10^−7^	2.64546 × 10^−5^	0.684464	0.753762	0.604342	0.686201958
0.0246	7.14 × 10^−4^	0.0081609	0.00023161	0.0014	3.35 × 10^−5^	0.069	2.10 × 10^−3^	1.34 × 10^−5^	3.87 × 10^−6^	5.65 × 10^−7^	3.33327 × 10^−5^	0.622601	0.652037	0.596173	0.692355618
0.036068	0.0011965	0.008198	0.00021169	0.0019	2.34 × 10^−5^	0.0411	1.50 × 10^−3^	1.94 × 10^−5^	4.09 × 10^−6^	5.24 × 10^−7^	4.20134 × 10^−5^	0.715567	5.78E‐01	3.70E‐01	0.570750885
0.016987	0.00049958	0.0154	3.18 × 10^−4^	0.0020325	5.28 × 10^−5^	0.1282	1.80 × 10^−3^	1.42 × 10^−5^	4.51 × 10^−6^	7.67 × 10^−7^	3.74584 × 10^−5^	0.50934	0.602626	0.668053	0.410699099
0.026419	0.0014162	0.010584	0.00040763	0.0025	6.87 × 10^−5^	0.1289	6.00 × 10^−3^	2.06 × 10^−5^	5.6 × 10^−6^	1.09× 10^−6^	5.19003 × 10^−5^	9.60E‐01	8.37E‐01	6.57E‐01	1.16E+00
0.017183	0.00049092	0.0134	3.64 × 10^−4^	0.001852	5.37 × 10^−5^	0.0389	1.40 × 10^−3^	1.27 × 10^−5^	5.26 × 10^−6^	8.8 × 10^−7^	3.80868 × 10^−5^	0.525732	0.685839	0.665733	5.75E‐01
0.0315	0.001	0.012232	0.00032292	0.0022	5.52 × 10^−5^	0.0813	2.40 × 10^−3^	1.94 × 10^−5^	5.55 × 10^−6^	9.53 × 10^−7^	4.90595 × 10^−5^	6.39E‐01	6.19E‐01	6.03E‐01	6.01E‐01
0.017959	6.98 × 10^−4^	0.015092	0.00042303	0.0038968	9.61 × 10^−5^	#N/A	#N/A	1.55 × 10^−5^	6.1 × 10^−6^	1.35× 10^−6^	4.84464 × 10^−5^	0.661484	0.697177	0.661788	#N/A
0.020511	0.00062309	0.01192	2.93 × 10^−4^	0.0026296	5.50 × 10^−5^	0.063	1.70 × 10^−3^	1.13 × 10^−5^	4.15 × 10^−6^	7.83 × 10^−7^	3.19772 × 10^−5^	6.47E‐01	6.58E‐01	6.06E‐01	5.99E‐01
#N/A	#N/A	0.016338	0.00051127	0.0040954	9.21 × 10^−5^	0.1814	0.0029	2.78 × 10^−5^	7.12 × 10^−6^	1.12× 10^−6^	6.60636 × 10^−5^	#N/A	0.749387	0.679452	0.418859263
0.015329	0.00040633	0.0095	2.46 × 10^−4^	0.0018	4.65 × 10^−5^	0.0606	2.10 × 10^−3^	1.12 × 10^−5^	3.47 × 10^−6^	6.39 × 10^−7^	3.0135 × 10^−5^	0.48941	0.677801	0.68533	0.776993825
0.023507	0.0009822	0.0166	6.32 × 10^−4^	#N/A	#N/A	0.1624	3.60 × 10^−3^	2.68 × 10^−5^	7.93 × 10^−6^	1.54× 10^−6^	7.11838 × 10^−5^	0.618419	0.870786	#N/A	0.529405911
0.044246	0.0010526	0.022661	0.00057132	0.005318	0.00014875	0.2844	0.0052	3.46 × 10^−5^	1.04 × 10^−5^	2.17× 10^−6^	9.28571 × 10^−5^	0.425341	0.589727	0.691871	0.505942362
0.0406	0.001	0.0193	5.20 × 10^−4^	0.0049	1.03 × 10^−4^	0.1927	3.80 × 10^−3^	3.13 × 10^−5^	8.67 × 10^−6^	1.52× 10^−6^	7.94691 × 10^−5^	0.443629	0.636102	0.594976	0.485527337
0.015473	0.00063677	0.014016	0.0003858	0.0022712	6.64 × 10^−5^	0.0859	2.00 × 10^−3^	2.12 × 10^−5^	5.36 × 10^−6^	8× 10^−7^	5.06068 × 10^−5^	0.555551	0.703879	0.779386	0.479621878
0.0139	3.74 × 10^−4^	0.0088	3.19 × 10^−4^	0.0024	7.25 × 10^−5^	0.0472	1.10 × 10^−3^	1.2 × 10^−5^	4.83 × 10^−6^	8.77 × 10^−7^	3.58948 × 10^−5^	0.458735	0.772895	0.789826	0.422547708
0.0162	8.40 × 10^−4^	0.0149	4.00 × 10^−4^	0.0048	1.47 × 10^−4^	0.0771	0.0027	2.3 × 10^−5^	7.92 × 10^−6^	1.76 × 10^−6^	6.56801 × 10^−5^	0.687587	0.581811	0.802468	0.599914924
0.017887	5.19 × 10^−4^	0.0083	2.20 × 10^−4^	0.0022	5.67 × 10^−5^	0.0582	0.0017	1.5 × 10^−5^	4.9 × 10^−6^	8.89 × 10^−7^	4.04667 × 10^−5^	0.500986	0.544678	0.640988	0.553871201

												Mean Damping ratio			
												Proximal	Middle	Distal	Abduction
												0.590567	6.73E‐01	0.654647	0.583788529
											Standard deviation	0.213799	0.10548	0.161225	0.204191306

**TABLE 4:** Little finger mean parameter estimate values for all degrees of freedom.

Little															
MCP flexion		PIP flexion		DIP flexion		Abduction		Moment of inertia (Kg m^2^/rad)				Damping ratio			
K (Nm/rad)	B (Nms/rad)	K (Nm/rad)	B (Nms/rad)	K (Nm/rad)	B (Nms/rad)	K (Nm/rad)	B (Nms/rad)	Proximal	Middle	Distal	Abduction	Proximal	Middle	Distal	Abduction
0.027405	5.95 × 10^−4^	0.0072	2.33 × 10^−4^	0.0016	5.24 × 10^−5^	0.0148	1.30 × 10^−3^	9.59 × 10^−6^	2.88 × 10^−6^	7.65 × 10^−7^	2.64281 × 10^−5^	5.80E‐01	8.07E‐01	7.49E‐01	1.04E+00
0.0082	2.49 × 10^−4^	0.0025	7.91 × 10^−5^	#N/A	#N/A	#N/A	#N/A	4.38 × 10^−6^	1.32 × 10^−6^	4.57 × 10^−7^	1.25615 × 10^−5^	0.657458	0.689397	#N/A	#N/A
0.011602	0.00047891	0.0044	1.57 × 10^−4^	1.30E‐03	5.13 × 10^−5^	0.0393	1.50 × 10^−3^	6.14 × 10^−6^	2.21 × 10^−6^	6.6 × 10^−7^	1.89962 × 10^−5^	0.897513	0.793944	0.875341	0.86802441
0.0053	2.04 × 10^−4^	#N/A	#N/A	#N/A	#N/A	0.0314	6.64 × 10^−4^	3.39 × 10^−6^	1.62 × 10^−6^	4.15 × 10^−7^	1.15628 × 10^−5^	0.760113	#N/A	#N/A	0.55105303
0.0116	4.23 × 10^−4^	0.0046	1.58 × 10^−4^	9.38 × 10^−4^	3.44 × 10^−5^	0.0093	4.15 × 10^−4^	6.61 × 10^−6^	2.39 × 10^−6^	5.76 × 10^−7^	1.97462 × 10^−5^	0.764093	0.751684	0.7401	0.484607589
0.0052	1.83 × 10^−4^	#N/A	#N/A	5.77 × 10^−4^	1.97 × 10^−5^	0.0137	5.68 × 10^−4^	3.82 × 10^−6^	1.19 × 10^−6^	2.97 × 10^−7^	1.05011 × 10^−5^	0.647158	#N/A	0.753078	0.749178136
0.0137	2.51 × 10^−4^	0.0035868	7.90 × 10^−5^	0.0012	2.79 × 10^−5^	0.0255	1.20 × 10^−3^	5.77 × 10^−6^	1.74 × 10^−6^	4.93 × 10^−7^	1.60917 × 10^−5^	0.446871	0.499128	0.573064	0.936657455
0.01309	0.00031029	0.0032	6.44 × 10^−5^	0.0012	2.72 × 10^−5^	0.0122	4.31 × 10^−4^	4.48 × 10^−6^	1.29 × 10^−6^	4.37 × 10^−7^	1.26047 × 10^−5^	0.640801	0.50139	0.593695	0.549019898
0.0129	2.61 × 10^−4^	0.0057	1.37 × 10^−4^	1.30 × 10^−3^	3.19 × 10^−5^	0.0401	6.15 × 10^−4^	4.48 × 10^−6^	1.7 × 10^−6^	4.62 × 10^−7^	1.3559 × 10^−5^	0.54206	0.693439	0.649521	0.41702966
0.0099	3.10 × 10^−4^	0.0027	6.48 × 10^−5^	0.002	4.52 × 10^−5^	0.0473	1.10 × 10^−3^	5.56 × 10^−6^	1.3 × 10^−6^	8.02 × 10^−7^	1.59788 × 10^−5^	0.661542	0.547732	0.564748	0.632645815
0.0123	3.88 × 10^−4^	#N/A	#N/A	0.0011674	3.31 × 10^−5^	0.0366	9.39 × 10^−4^	5.47 × 10^−6^	1.4 × 10^−6^	4.45 × 10^−7^	1.43685 × 10^−5^	0.747967	#N/A	0.724833	0.647658971
0.0142	5.26 × 10^−4^	0.0034	9.61 × 10^−5^	8.22 × 10^−4^	2.31 × 10^−5^	0.0101	6.86 × 10^−4^	7.24 × 10^−6^	1.5 × 10^−6^	4.95 × 10^−7^	1.7313 × 10^−5^	8.20E‐01	0.67229	5.72E‐01	8.20E‐01
0.015349	0.00054434	0.0064	1.51 × 10^−4^	0.0028	6.49 × 10^−5^	0.0547	1.70 × 10^−3^	1.04 × 10^−5^	2.75 × 10^−6^	1.13 × 10^−6^	2.96937 × 10^−5^	0.681916	0.568668	0.577392	0.666950254
0.012669	0.0003663	0.0063163	1.43 × 10^−4^	0.001665	4.03 × 10^−5^	0.0238	1.20 × 10^−3^	5.99 × 10^−6^	2.06 × 10^−6^	7.25 × 10^−7^	1.82437 × 10^−5^	6.65E‐01	6.26E‐01	5.81E‐01	9.11E‐01
0.014196	0.00077897	0.0069	1.82 × 10^−4^	0.0034367	7.80 × 10^−5^	0.0766	1.30 × 10^−3^	1.21 × 10^−5^	2.88 × 10^−6^	9.02 × 10^−7^	3.0358 × 10^−5^	0.94093	0.64439	7.00E‐01	0.426248307
0.0062	1.65 × 10^−4^	0.0016	4.19 × 10^−5^	4.80 × 10^−4^	1.55 × 10^−5^	0.0204	3.93 × 10^−4^	3.86 × 10^−6^	9.66E‐07	3.87 × 10^−7^	1.04059 × 10^−5^	5.32E‐01	5.33E‐01	5.68E‐01	4.27E‐01
0.013802	0.00041174	0.0049	1.18 × 10^−4^	2.20 × 10^−3^	4.11 × 10^−5^	#N/A	#N/A	8.79 × 10^−6^	2.03 × 10^−6^	8.53 × 10^−7^	2.31846 × 10^−5^	0.591137	0.591251	0.474787	#N/A
0.032	8.72 × 10^−4^	0.010212	1.83 × 10^−4^	0.0044	8.96 × 10^−5^	0.0671	1.70 × 10^−3^	1.5 × 10^−5^	3.21 × 10^−6^	1.24 × 10^−6^	3.75951 × 10^−5^	6.29E‐01	5.05E‐01	6.06E‐01	5.35E‐01
0.017871	6.17 × 10^−4^	0.007412	1.75 × 10^−4^	0.0020557	5.31 × 10^−5^	0.0746	1.70 × 10^−3^	1.34 × 10^−5^	2.84 × 10^−6^	7.49 × 10^−7^	3.12352 × 10^−5^	0.631203	0.60224	0.676726	0.556835774
0.0083	3.77 × 10^−4^	0.0051	1.34 × 10^−4^	0.0018	4.73 × 10^−5^	0.053	1.30 × 10^−3^	6.45 × 10^−6^	2.05 × 10^−6^	5.7 × 10^−7^	1.84887 × 10^−5^	0.814509	0.654444	0.73815	0.656632166
0.009897	4.62 × 10^−4^	0.0038385	9.10 × 10^−5^	0.0015	3.23 × 10^−5^	0.0389	8.30 × 10^−4^	6.18 × 10^−6^	1.37 × 10^−6^	4.78 × 10^−7^	1.5506 × 10^−5^	0.933159	0.628302	0.604014	0.534294906
0.0062	3.33 × 10^−4^	0.0052	1.41 × 10^−4^	0.0026	7.06 × 10^−5^	0.0628	1.30 × 10^−3^	8.04 × 10^−6^	2.61 × 10^−6^	9 × 10^−7^	2.36221 × 10^−5^	0.746257	0.604495	0.729704	0.53367174
0.0104	2.70 × 10^−4^	0.0043	9.61 × 10^−5^	0.0016	3.69 × 10^−5^	0.0228	8.45 × 10^−4^	6.75 × 10^−6^	1.54 × 10^−6^	5.36 × 10^−7^	1.6928 × 10^−5^	0.509981	0.589613	0.630004	0.680380968

												Mean Damping ratio			
												Proximal	Middle	Distal	Abduction
												6.89E‐01	0.625206	0.651465	0.648713757
											Standard Deviation	0.132895	0.231224	0.207972	0.25300335

One particularly interesting outcome of the study, as can be seen from the raw data in Figures 4, 5, 6, 7, 8, is that the free response of the segments, for all of the degrees of freedom, oscillates before reaching its steady state. This behaviour is characteristic of underdamped motion. Thus, the average damping ratio was calculated for each segment for each degree of freedom using the following expression:

(24) $$\zeta \;=\frac{B_{i}}{2I_{i}\sqrt{\frac{K_{i}}{I_{i}}}}\;$$

In Table 5 the mean damping ratio values are provided for all of the trials.

**TABLE 5:** Mean values of the damping ratio for each segment in each degree of freedom alongside their standard deviation (SD).

Movement	Index damping ratio (SD)	Middle damping ratio (SD)	Ring damping ratio (SD)	Little damping ratio (SD)
MCP flexion	0.562 (0.07)	0.614 (0.15)	0.591 (0.21)	0.689 (0.13)
PIP flexion	0.665 (0.08)	0.688 (0.09)	0.673 (0.1)	0.625 (0.23)
DIP flexion	0.687 (0.1)	0.681 (0.15)	0.655 (0.16)	0.652 (0.21)
Abduction	0.574 (1.1)	0.63 (0.26)	0.584 (0.2)	0.649 (0.25)

## 9 DISCUSSION

The torsional spring and damper constants for all the degrees of freedom for the index through to the little finger segments were estimated. Comparison between the values determined here and those found in the literature can only be done for the torsional spring constant of the DIP and MCP joints for the flexion/extension movements of the index finger. In the work presented in [21] a mean and standard deviation for the torsional spring constant for the middle segment were obtained using a robot that flexed the DIP joint of the index finger while the MCP joint of the same finger was held at either 0 or 60 degrees. In the experiment presented in [21] the authors used the same wrist orientation for the flexion/extension movement as presented here. From the data presented in [21] the extension stiffness value from their trial‐to‐trial reproducibility study is compared to the mean value of all the torsional spring constants of the PIP flexion of the index finger from Table 1. The extension value given in [21] was used because the data that were fitted to the free response equations correspond to the extension of the segments returning to their steady state.

In Table 6, the parameter values from this study and those found in [21] can be seen. The percentage difference between the two values is 29.08%. Even though the percentage difference is relatively high, this can be attributed to the difference in sample size and anthropometrical variation between the two studies. Another possible explanation for the percentage difference can be attributed to the Coriolis forces that come from the relative movement of the proximal and distal phalanges during the experiment. However, given the high standard deviation from our study, the mean value from [21] is within the boundary set by the standard deviation.

**TABLE 6:** Mean value with standard deviation (SD) of the torsional spring constant from all trials of the PIP flexion as shown in Table 1 and the extension stiffness determined from the trial‐to‐trial study in [21].

Method	K (N m/rad)	SD (N m/rad)
This study	0.0144	0.0064
[21]	0.0193	0.0022

In the work presented in [25], the authors reported the passive MCP joint stiffness that was determined with two different methods using a standard stiffness measurement device and a soft robotic actuator. A similar wrist orientation was assumed in their study as that used in this paper. In the following table, the mean values together with the standard deviation (SD) for the results from [25] and the mean of the results presented in this paper are provided for comparison.

In Table 7, the parameter values from this study and those found in [21] can be seen. The percentage differences between the two different methods in [25] and this study are 12.18% for the soft robotic actuator and 6.61% for the stiffness measurement device. The percentage errors for the MCP joint are smaller compared to the PIP and this can be attributed to the fact that the contribution of the Coriolis forces on the proximal phalanx from the relative motion of the second metacarpal is diminished. However, from the previous two tables, it is apparent that the method presented in this paper provides results that are in good agreement with other methods found in the literature. This study has been shown to agree with different experiments for estimating the torsional spring constant of the passive moment of the fingers. The values found in Tables 1, 2, 3, 4 have the potential to be used as a reference for the values of the passive moment contributions. The main advantage of this study is the reproducibility of the experimental procedure as it does not rely on any robotic actuators compared to those found in [21, 25]. Potential improvements to the experimental protocol might be to include a medical clamp to fix the elbow and wrist in place, which reduces fatigue for the participant, and potentially the use of an anesthetic agent that can numb the sensation on the fingers. The latter is believed to allow the involuntary movement of the free response to be exactly that, involuntary, without the participants having any reflex control over the moving segment.

**TABLE 7:** Mean value with standard deviation (SD) of the torsional spring constant from all trials of the MCP flexion as shown in Table 1 and the extension stiffness determined from the data available at [25].

Method	K (N m/rad)	SD (N m/rad)
Soft robotic actuator [25]	0.0324	0.0058
Stiffness measurement device [25]	0.0391	0.0057
This study	0.0366	0.0127

Another interesting outcome of the study is the observed variation in the parameters across the recruited population. This is in contrast to the results presented in [4] where the authors assumed the same spring and damper constant values for all fingers for all participants. Given the extent to which these parameters vary, it is therefore important that appropriate scaling functions are derived in order to allow subject specific estimation of the passive moment parameters to be determined based on anthropometric data. On this basis, scaling functions between the torsional spring and damper parameters and the anthropometric values obtained were also determined. Linear fits between the estimated parameters and different anthropometrical measurements were examined. These measurements were the segment length (*sl*), segment radius (*sr*), the sum of the palm length (*pl*), palm breadth (*pb*), palm width (*pw*), the product between pl and pb as suggested in [26], the product between the hand length (*hl*), which is the sum of the palm length and the sum of the lengths of all the segments of the middle finger and *pb*. Lastly, the product of segment length and segment radius, the sum of the individual segment lengths (*Ssl*), the sum of the individual radii (*Ssr*), and the sum of the individual products of segment lengths and radii (*SLR*) were calculated (the last three were considered for the abduction movement only). For example, the *SLR*, *Ssl*, and *Ssr* for the index finger are given by, respectively:

(25) $$\left\{\begin{array}{c} SL\;R_{\mathrm{index}}=\sum_{i=1}^{3}sl_{\mathrm{index},i}\times sr_{\mathrm{index},i}\;\\ Ss\;l_{\mathrm{index}}=\sum_{i=1}^{3}sl_{\mathrm{index},i}\;\\ Ss\;r_{\mathrm{index}}=\sum_{i=1}^{3}sr_{\mathrm{index},i}\; \end{array}\right.$$

The normalized inverse of the standard deviation of the parameters was used as a weighting factor in the fitting process. Parameters with a low standard deviation had a higher weighting value than those with a high standard deviation. Fits that had the highest *R* ^2^ value were chosen. The scaling functions can be seen alongside the *R* ^2^ value below. The anthropometric parameters are given in, mm and the torsional spring and damper constants are in Nm/rad and Nms/rad, respectively. In Table 8, the fitting coefficients with their associated *R* ^2^ value, alongside the equations used for scaling the parameters, can be found.

**TABLE 8:** Table with the scaling functions for the torsional spring and damper constants for all the degrees of freedom for the index through to the little finger.

	Torsional spring scaling equation (N m/rad)	*R* ^2^	Torsional damper scaling equation (N ms/rad)	*R* ^2^
Index Proximal segment (mm)	$-0.041+0.0002\times sl_{\mathrm{index},\mathrm{prox}}\times sr_{\mathrm{index},\mathrm{prox}}$	0.89	$-0.0011+5.78\times 10^{-6}\times sl_{\mathrm{index},\mathrm{prox}}\times sr_{\mathrm{index},\mathrm{prox}}$	0.88
Index middle segment (mm)	$-0.02+0.00015\times sl_{\mathrm{index},\mathrm{middle}}\times sr_{\mathrm{index},\mathrm{middle}}$	0.79	$-0.0007+4.72\times 10^{-6}\times sl_{\mathrm{index},\mathrm{middle}}\times sr_{\mathrm{index},\mathrm{middle}}$	0.81
Index distal segment (mm)	$-0.003+3.24\times 10^{-5}\times sl_{\mathrm{index},\mathrm{distal}}\times sr_{\mathrm{index},\mathrm{distal}}$	0.72	$-0.0001+9.5\times 10^{-7}\times sl_{\mathrm{index},\mathrm{distal}}\times sr_{\mathrm{index},\mathrm{distal}}$	0.84
Index Abduction (mm)	$-0.16+0.00036\times SLR_{\mathrm{index}}$	0.74	$-0.0043+9.12\times 10^{-6}\times SLR_{\mathrm{index}}$	0.74
Middle Proximal segment (mm)	$-0.036+0.0002\times sr_{\mathrm{middle},\mathrm{prox}}\times sl_{\mathrm{middle},\mathrm{prox}}$	0.68	$-0.0011+6.75\times 10^{-6}\times sr_{\mathrm{middle},\mathrm{prox}}\times sl_{\mathrm{middle},\mathrm{prox}}$	0.72
Middle middle segment (mm)	$-0.026+0.0002\times sr_{\mathrm{middle},\mathrm{middle}}\times sl_{\mathrm{middle},\mathrm{middle}}$	0.63	$-0.0009+5.27\times 10^{-6}\times sr_{\mathrm{middle},\mathrm{middle}}\times sl_{\mathrm{middle},\mathrm{middle}}$	0.8
Middle distal segment (mm)	$-0.0042+3.61\times 10^{-5}\times sr_{\mathrm{middle},\mathrm{distal}}\times sl_{\mathrm{middle},\mathrm{distal}}$	0.58	$-0.0001+1.14\times 10^{-6}\times sr_{\mathrm{middle},\mathrm{distal}}\times sl_{\mathrm{middle},\mathrm{distal}}$	0.86
Middle Abduction (mm)	$-0.285+2.676\times 10^{-5}\times hl\times hb$	0.52	$-0.0085+7.83\times 10^{-7}\times hl\times hb\;$	0.65
Ring Proximal segment (mm)	$-0.03+0.0015\times sl_{\mathrm{ring},\mathrm{prox}}$	0.52	$-0.0012+5.62\times 10^{-5}\times sl_{\mathrm{ring},\mathrm{prox}}$	0.67
Ring middle segment (mm)	$-0.018+0.00014\times sl_{\mathrm{ring},\mathrm{middle}}\times sr_{\mathrm{ring},\mathrm{middle}}$	0.66	$-0.00054+3.928\times 10^{-6}\times sl_{\mathrm{ring},\mathrm{middle}}\times sr_{\mathrm{ring},\mathrm{middle}}$	0.65
Ring distal segment (mm)	$-0.0033+3.37\times 10^{-5}\times sl_{\mathrm{ring},\mathrm{distal}}\times sr_{\mathrm{ring},\mathrm{distal}}$	0.77	$-0.00013+1.12\times 10^{-6}\times sl_{\mathrm{ring},\mathrm{distal}}\times sr_{\mathrm{ring},\mathrm{distal}}$	0.93
Ring Abduction (mm)	$-0.536+0.0069\times Ssl_{\mathrm{ring}}$	0.67	$-0.0094+0.00013\times Ssl_{\mathrm{ring}}$	0.62
Little Proximal segment (mm)	$-0.0218\;+0.0012\times \;sl_{\text{little},\text{prox}}$	0.46	$-0.00092+4.788\times 10^{-5}\times sl_{\mathrm{little},\mathrm{prox}}$	0.72
Little middle segment (mm)	$-0.0064+7.69\times 10^{-5}\times sl_{\mathrm{little},\mathrm{middle}}\times sr_{\mathrm{little},\mathrm{middle}}$	0.76	$-0.00019+2.123\times sl_{\mathrm{little},\mathrm{middle}}\times sr_{\mathrm{little},\mathrm{middle}}$	0.83
Little distal segment (mm)	$-0.0037+3.70\times 10^{-5}\times sl_{\mathrm{little},\mathrm{distal}}\times sr_{\mathrm{little},\mathrm{distal}}$	0.86	$-7.66\times 10^{-5}+8.04\times 10^{-6}\times sl_{\mathrm{little},\mathrm{distal}}\times sr_{\mathrm{little},\mathrm{distal}}$	0.86
Little Abduction (mm)	−0.1074 + 0.0066 × $Ssr_{\mathrm{little}}$	0.36	$-0.0011\;+\;4.073\times 10^{-6}\;SLR_{\mathrm{little}}$	0.5

With the equations shown in Table 8 the torsional spring and damper parameters of the passive moment can be determined for any individual.

As mentioned, a particularly interesting finding of this study was that the free response of the finger segments is underdamped for all degrees of freedom. This is evident from the mean damping ratio values as shown in Table 5. This characteristic movement of the segments has not been reported previously in the literature. From the type of experiment performed, it is evident that these values are characteristic of the underlying structure of the human finger segments. Since this is an involuntary movement, it provides a glimpse of the passive structure and a quantification of the passive moment generated at each joint. An underdamped motion is characterized by lower force/torque production on a system compared to other types of free response, hence the characteristic oscillation about its steady state. However, since this has seemingly not been reported elsewhere in the literature, the conclusion is drawn here that, during voluntary motion of the finger segments, the passive moment increases and becomes either critically damped or overdamped. Potentially, this means that the passive moment components are dynamic in nature rather than static scalar parameters as considered previously in the literature.

## 10 CONCLUSION

In this paper, a novel mathematical description of the equations of motion of human finger segments based on Lagrangian mechanics and cylindrical approximations of the segments of human digits has been derived. A linear second‐order differential equation approximation to the underlying equations of motion for each degree of freedom is established, and parameter estimation has been performed using this model for the free response movement. An experiment has been designed that corresponds to the free response movement, and a novel marker set has also been applied to obtain corresponding angular data. It has been shown that the free response of the finger segments is underdamped for all the degrees of freedom and scaling functions between the torsional spring and damper parameters and subject anthropometry have been derived. The intention is that this model will be used to support parameter estimation during voluntary movement which can be controlled by a musculoskeletal model with surface electromyography (sEMG) as its actuator. This has the potential to further our understanding of how passive moments change, if at all, during voluntary movement controlled by the central nervous system. Moreover, the model has the potential to be used in the design of a neuroprosthesis as a means to incorporate feedback control for joint state estimation from sEMG data and haptic feedback mechanisms for both partial and complete hand amputees.

## AUTHOR CONTRIBUTIONS

All authors contributed to writing the paper. Panagiotis Tsakonas developed the methodology, analysed the data, wrote the MATLAB code, ran the experiments, and, with the help of Evans Neil and Michael J. Chappell, designed the experimental protocol of the study. Joseph Hardwicke provided his expertise in hand anatomy and palpation techniques for accurately measuring segment dimensions. Michael J. Chappell, Joseph Hardwicke and Evans Neil acquired the funding for this project. Evans Neil and Michael J. Chappell were the main project supervisors. Joseph Hardwicke and Michael J. Chappell were the main project administrators.

## CONFLICT OF INTEREST STATEMENT

The authors declare no conflicts of interest.

## Data Availability

The data for the values of spring and damper constants alongside the moments of inertia are available from the corresponding author upon reasonable request. Motion capture alongside segment length and radii data are not available due to ethical restrictions.
